# TATA binding protein associated factor 3 (TAF3) interacts with p53 and inhibits its function

**DOI:** 10.1186/1471-2199-9-57

**Published:** 2008-06-12

**Authors:** Orsolya Bereczki, Zsuzsanna Ujfaludi, Norbert Pardi, Zita Nagy, Laszlo Tora, Imre M Boros, Eva Balint

**Affiliations:** 1 Department of Biochemistry and Molecular Biology, University of Szeged, Szeged, Hungary; 2 Institute of Biochemistry, Biological Research Center, Szeged, Hungary; 3 Institut de Genetique et de Biologie Moleculaire et Cellulaire (IGBMC), UMR 7104 CNRS, ULP, INSERM U.596, Illkirch, Strasbourg, France; 4 BayGen Institute, Bay Zoltan Foundation for Applied Research, Szeged, Hungary

## Abstract

**Background:**

The tumour suppressor protein p53 is a sequence specific DNA-binding transcription regulator, which exerts its versatile roles in genome protection and apoptosis by affecting the expression of a large number of genes. In an attempt to obtain a better understanding of the mechanisms by which p53 transcription function is regulated, we studied p53 interactions.

**Results:**

We identified BIP2 (Bric-à-brac interacting protein 2), the fly homolog of TAF3, a histone fold and a plant homeodomain containing subunit of TFIID, as an interacting partner of *Drosophila melanogaster *p53 (Dmp53). We detected physical interaction between the C terminus of Dmp53 and the central region of TAF3 both in yeast two hybrid assays and *in vitro*. Interestingly, DmTAF3 can also interact with human p53, and mammalian TAF3 can bind to both Dmp53 and human p53. This evolutionarily conserved interaction is functionally significant, since elevated TAF3 expression severely and selectively inhibits transcription activation by p53 in human cell lines, and it decreases the level of the p53 protein as well.

**Conclusion:**

We identified TAF3 as an evolutionarily conserved negative regulator of p53 transcription activation function.

## Background

The p53 tumour suppressor protein orchestrates cellular response to genotoxic stress such as DNA damage, activated oncogenes and other metabolic changes [1]. In unstressed cells, p53 is kept at a low level through the action of various ubiquitin ligases [2], while cellular stress results in the stabilization of p53 and its activation as a transcription factor. Although activated p53 is able to induce cell death via transcription-independent ways [3], importantly, it influences the transcription of a large number of genes, that in turn promote cell cycle arrest, DNA repair or apoptosis [4]. The multiple mechanisms that stabilize and activate p53 include post-translational modifications and interactions with different co-factors. For example, upon DNA damage resulting from ionizing radiation p53 is phosphorylated by ATM/ATR and Chk1/Chk2 kinases [5,6]. Activation of p53 upon UV radiation is less well understood, among other mechanisms, it may involve p33ING2 that increases the transcriptional-transactivation activity of p53 by enhancing its acetylation [7]. Interestingly, p33ING2 is a plant homeodomain (PHD) containing protein [7].

In vertebrates, the p53 gene has two other homologs, p63 and p73. Both of them express several isoforms through alternative promoter usage and alternative splicing, thereby generating a multitude of p53-related proteins that participate in the regulation of p53 transcriptional activity [8,9]. The only *Drosophila *homolog of p53, Dmp53, shares limited conservation with mammalian p53 at the sequence level, yet the two proteins are similar in domain structure and their residues critical for DNA binding are well preserved [10-12]. Importantly, Dmp53 is able to bind to human p53 recognition sites and activate transcription through these sites. *Dmp53 *null mutant flies are viable under normal circumstances, however, they show genomic instability and sensitivity to ionizing and UV radiation [13-15]. Overexpression of wild type p53 or Dmp53 in flies induces cell death, while expression of dominant negative Dmp53 inhibits apoptosis induced by genotoxic stimuli [10,12,16]. In response to DNA damage, Dmp53 induces DNA repair or programmed cell death by activating the expression of its target genes [15,17,18]. Activation of Dmp53 apparently does not involve stabilization of the protein, but similarly to its human counterpart, Dmp53 is phosphorylated by Chk2 and this modification is necessary for the induction of Dmp53 dependent apoptosis [18,19].

Activation domains of various transcription factors are able to interact directly with TATA binding protein (TBP) associated factors (TAFs), thus TAFs are thought to serve as bridging factors between sequence-specific transcription factors and the basal transcriptional machinery [20]. The p53 protein has also been found to interact with several components of the TFIID complex. The N-terminal transcriptional activation domain of p53 binds to TAF1, TAF6 and TAF9 [21,22]. While binding of TAF9 results in stabilization and activation of p53 [23,24], TAF1 phosphorylates p53 and promotes its degradation [25,26]. These interactions are evolutionarily conserved, since *Drosophila *TAFs and TBP can functionally interact with human p53 in heterolog systems [20].

DmTAF3/BIP2 (Bric-à-brac Interacting Protein 2) was first identified in a yeast two hybrid screen as an interacting partner of BAB1 and BAB2 (Bric à brac 1 and 2) *Drosophila *transcription factors [27]. Also, both *Drosophila *and mammalian TAF3 were found to be subunits of the respective TFIID complexes and dimerization partners for TAF10 [28]. Interestingly, TAF3 is also a PHD containing protein [28]. Developmental regulation of TAF3 expression has been demonstrated [29] and it has been recently shown that differentiation of myoblasts to myotubes involves the replacement of a canonical TFIID complex by a TRF3(TBP2)-TAF3 complex [30]. This previously unrecognized switching of the core promoter recognition complexes during differentiation may allow cells to selectively turn on a cell type-specific transcription program.

In search for interacting partners of Dmp53, we identified *Drosophila *BIP2/DmTAF3. Unlike the previously identified p53-interacting TAFs, DmTAF3 interacts with the C terminus of Dmp53. The interaction is evolutionarily conserved, since we show that mammalian TAF3 can bind to both Dmp53 and human p53. Overexpression of TAF3 severely inhibits transcription activation by p53 in human cell lines and TAF3 decreases the level of the p53 protein as well. Thus, we identified TAF3 as an evolutionarily conserved novel regulator of p53 transcription activation function.

## Results

### Dmp53 interacts with BIP2/DmTAF3

**Figure 1:**
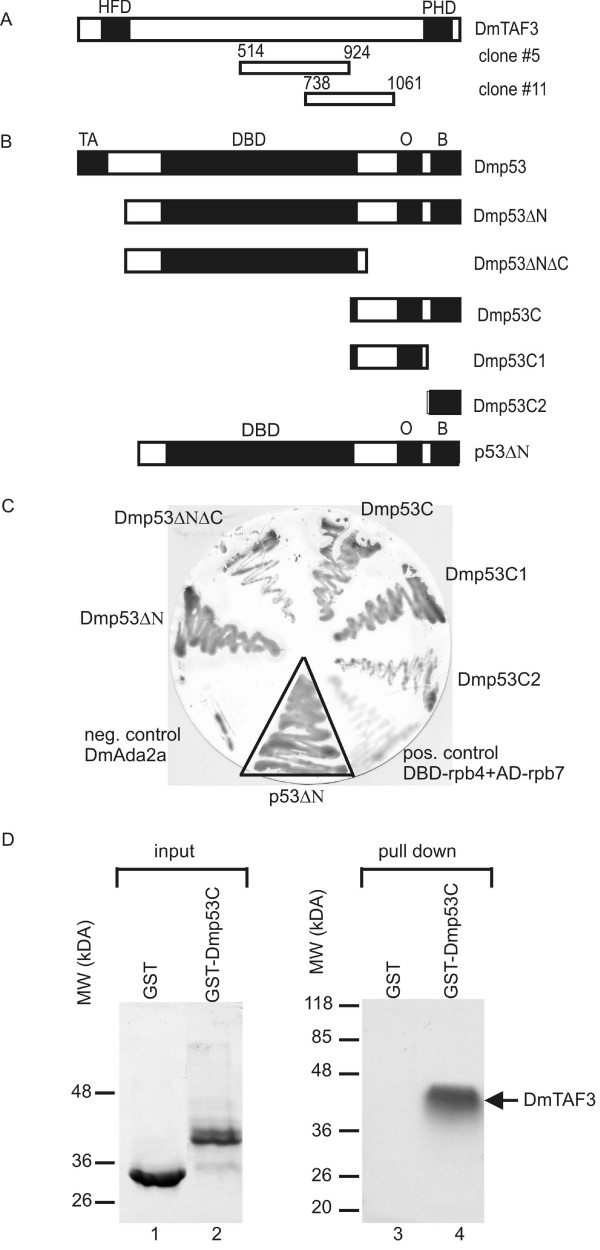
**Dmp53 interacts with DmTAF3**. (A) Full length DmTAF3 and the clones identified from Y2H screen as p53 interacting proteins (clone 5 and clone 11) are depicted. HFD, histone fold domain; PHD, plant homeodomain. (B) The various portions of Dmp53 and human p53 that were fused to lexA-DBD and used in Y2H experiments are depicted. TA, transactivation domain; DBD, DNA binding domain; O, oligomerization domain; B, basic regulatory domain. (C) The indicated lexA-DBD fusion constructs were co-introduced into yeast with DmTAF3 amino acids 514 to 924 fused to Gal4 activation domain (AD). Interacting proteins result in complementation of His-auxotrophy and induction of β-galactosidase activity, which was detected by filter assay. Negative control was DBD-ADA2A with the same DmTAF3 clone. Positive control was DBD-rpb4 with AD-rpb7. The p53ΔN-DmTAF3 interaction was examined on a separate filter with the same positive and negative controls, and the image was fitted into the figure. (D) Direct binding of DmTAF3 to Dmp53C *in vitro*. Coomassie stained gel of bacterially expressed GST (lane 1) and GST-Dmp53C (lane 2). DmTAF3 (aa 514–924) was expressed *in vitro *in the presence of ^3^H-labelled leucine and incubated with GST (negative control) or GST-Dmp53C. Glutathione-agarose bead bound proteins were eluted, electrophoretically separated and detected by autoradiography (lane3 and 4).

In order to identify Dmp53-interacting partners, we screened *Drosophila *embryonic cDNA library with the yeast two-hybrid (Y2H) method using Dmp53 fused to lexA DNA binding domain (DBD) as bait. Since Dmp53 contains a transcription activation domain at its N terminus, to avoid false activation, we used an N-terminally truncated version of Dmp53. Two of the identified positive clones contained partial cDNAs of the *Bric-à-Brac interacting protein *gene (*bip2*/CG2009), that encodes for a TBP-associated factor also called DmTAF3 [31]. A 1236 bp long clone (clone 5) encodes amino acids 514 to 924, while an 972 bp long clone (clone11) encodes amino acids 738 to 1061 of DmTAF3 (Fig. 1A) indicating that amino acids 738–924 are responsible for the interaction with Dmp53.

To determine which region of Dmp53 is necessary for the binding to DmTAF3, we tested various segments of Dmp53 (Fig. 1B) for interaction with one of the identified DmTAF3 clones (clone 5, Fig. 1A) in Y2H experiments. The DNA binding domain of Dmp53 showed weak interaction, and the C terminal part of Dmp53 showed strong interaction with DmTAF3 in the Y2H assay (Fig. 1C). In contrast, no interaction was observed between DmTAF3 and *Drosophila *ADA2A, a transcriptional co-activator/adaptor, indicating that the interaction between Dmp53 and DmTAF3 is specific. We tried to delineate the C-terminal binding region of Dmp53, and we found that the oligomerization domain alone interacted strongly, and also the basic regulatory domain alone interacted weakly with DmTAF3 (Fig. 1C). These data suggest that DmTAF3 interacts with Dmp53 at multiple binding sites.

To test whether the interaction between DmTAF3 and Dmp53 is direct, we performed an *in vitro *binding assay. We produced the polypeptide corresponding to amino acids 514 to 924 of DmTAF3 using rabbit reticulocyte *in vitro *transcription-translation system and bacterially expressed and purified GST-tagged Dmp53C (Fig. 1D lane 2). The identified portion of DmTAF3 bound to GST-Dmp53C, but not to GST (Fig. 1D lanes 3 and 4), suggesting that the interaction is specific and direct.

### The interaction between TAF3 and p53 is evolutionarily conserved

Despite the low sequence similarity between Dmp53 and human p53, human p53 has been observed before to interact with *Drosophila *TBP [20], and other *Drosophila *proteins that are also binding partners of Dmp53 [32]. Therefore, we were interested in whether DmTAF3 is able to interact with human p53. Since human p53 also contains transcription activation domain at its N-terminal region, to avoid false positives, we used N-terminally deleted p53 in Y2H assays (Fig. 1B). We detected strong interaction between DmTAF3 and human p53 protein (Fig. 1C). This result suggests that the amino acids responsible for interaction with DmTAF3 are conserved in human p53.

**Figure 2:**
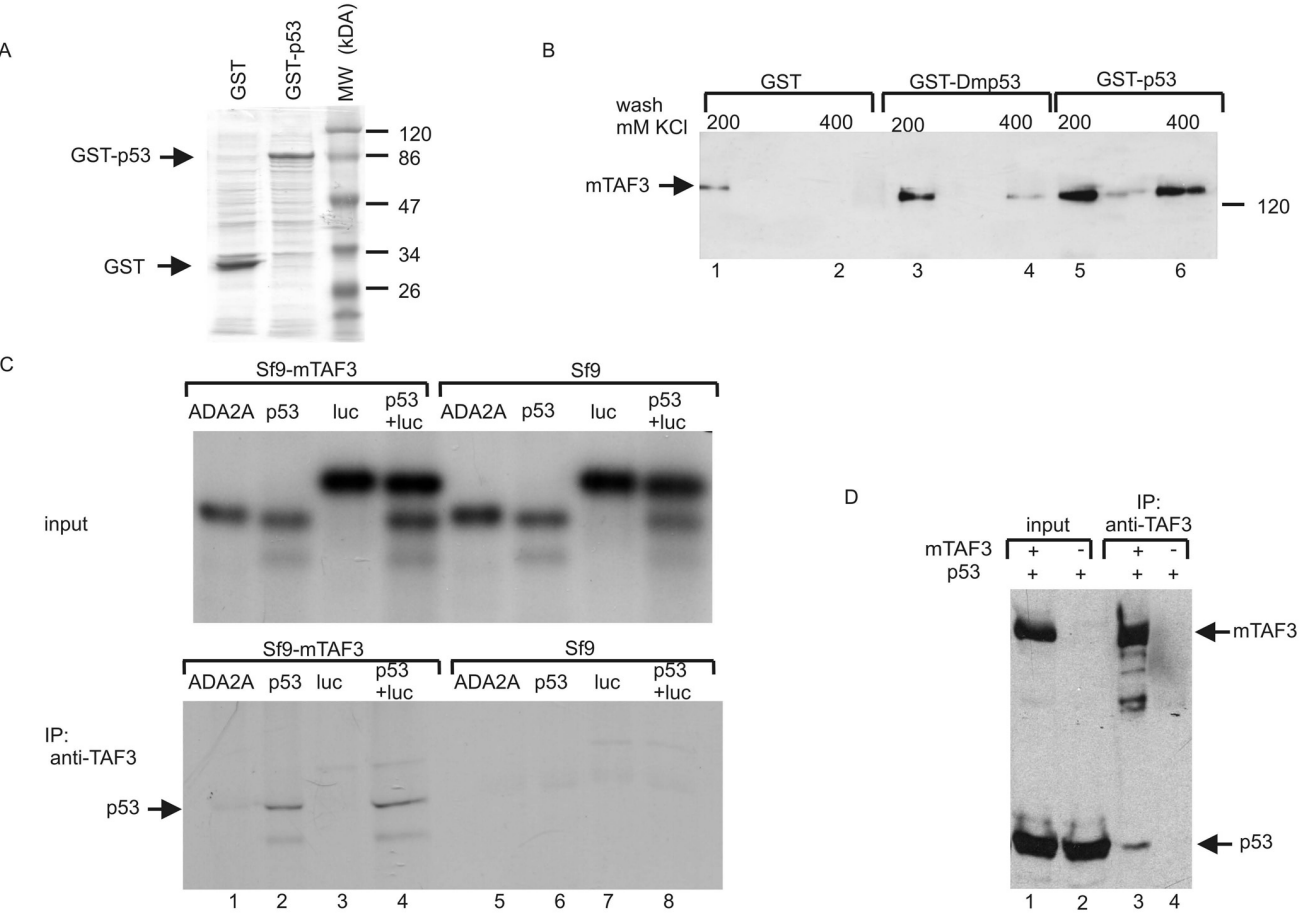
***In vitro *and *in vivo *binding of mTAF3 to Dmp53 and p53**. (A) GST and GST-p53 was expressed in bacteria as described in Methods, SDS-PAG was stained with Coomassie Brilliant Blue. (B) Mammalian TAF3 binds human p53 and Dmp53 as well. Cell extract from Sf9 cells overexpressing full length murine TAF3 was incubated with GST, GST-Dmp53C or GST-p53 bound to glutathione-agarose beads. Beads were washed with various concentrations of KCl as indicated above the lanes, then bound proteins were eluted and analysed with Western blotting using anti-TAF3 antibody. (C) TAF3 binds p53 *in vitro*. Drosophila ADA2A, human p53 and firefly luciferase were *in vitro *translated and transcribed using ^35^S-methionine. The proteins were then mixed with cell extract overexpressing mTAF3, an aliquot was taken for input control that was analysed by autoradiography (upper panel). Binding proteins were immunoprecipitated using anti-TAF3 antibody, and precipitated radioactive proteins were detected as above (lower panel). (D) p53 binds TAF3 *in vivo*. Plasmids overexpressing human p53 and mTAF3 (+) or empty vector (-) were co-transfected into 293T cells as indicated above the lanes. Complexes were immunoprecipitated using anti-TAF3 antibody and Western blot was done using anti-p53 and anti-TAF3 antibodies.

Next we tested whether the mammalian homolog of DmTAF3 would interact *in vitro *with p53 as well. To this end we expressed full length murine TAF3 (mTAF3) in Sf9 insect cells and produced GST, GST-Dmp53C or GST-p53 in bacteria (Fig. 1D and 2A). In these GST-pull-down assays mTAF3 bound to GST-Dmp53C and GST-p53 (Fig. 2B lanes 3–6). Note however, that mTAF3 showed a weak binding to GST alone under 200 mM salt washing conditions (Fig. 2B lane 1), but it bound specifically to GST-Dmp53C and GST-p53 at higher salt concentration (400 mM) (Fig. 2B lanes 4, 6) where it did not bind to the GST control any more (Fig. 2B lane 2). These data indicate that mTAF3 specifically interacts with p53, and also, although less strongly, with Dmp53 suggesting that the TAF3-p53 interaction is evolutionarily conserved.

To confirm the interaction in a reverse experiment, where we pull down TAF3, we translated full length human p53 and Drosophila ADA2A or firefly luciferase as controls *in vitro*, mixed them with cell extract containing overexpressed mTAF3, and immunoprecipitated mTAF3-containing complexes using an anti-TAF3 antibody. p53 co-precipitated with mTAF3, while ADA2A and luciferase did not (Fig. 2C lanes 1–3). In another control experiment, where we added p53 and luciferase proteins together to the cell extract containing overexpressed mTAF3, anti-TAF3 antibody co-immunoprecipitated p53 but not luciferase (Fig. 2C lane 4). Furthermore, when we added p53 to cell extract containing no mTAF3, p53 did not precipitate with the anti-TAF3 antibody (Fig. 2C lanes 6 and 8). These data confirm a specific interaction between p53 and mTAF3.

Next we investigated, whether the p53-TAF3 interaction occurs in human cells as well. To this end, we co-transfected plasmids expressing mTAF3 and p53 into 293T cells, then we immunoprecipitated TAF3-containing complexes with an anti-TAF3 antibody, and detected p53 by Western blotting. Under these conditions, a small amount of the overexpressed p53 co-precipitated with mTAF3 (Fig. 2D lane 3). In the control experiment, when we co-transfected p53 with an empty vector, no p53 precipitated with the anti-TAF3 antibody (Figure 2D lane 4), indicating the specificity of the TAF3 antibody. These data together demonstrate that p53 protein can interact with TAF3 in cells.

### TAF3 inhibits the transcription activation function of p53

**Figure 3:**
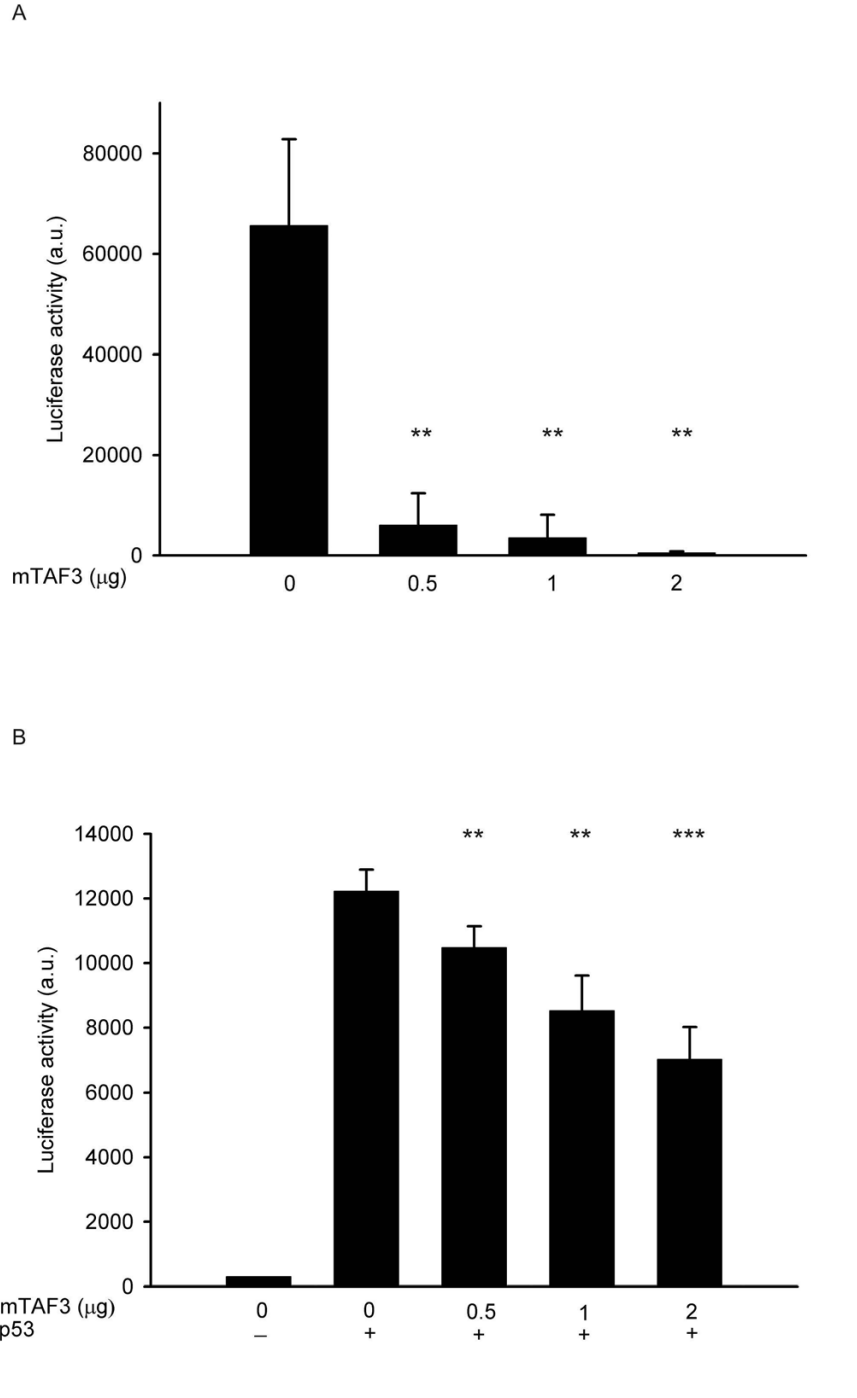
**Overexpression of mTAF3 strongly inhibits the transcriptional activity of p53**. (A) U2OS cells were transfected with a p53-responsive luciferase reporter construct and the indicated amounts of plasmid overexpressing mTAF3 (0, 0.5, 1, 2 μg) supplemented with empty vector to a total of 3 μg DNA. (B) HeLa cells were co-transfected with 0.5 μg plasmid overexpressing p53 where indicated by a +, and the same luciferase reporter as in A, and plasmid overexpressing mTAF3 (0, 0.5, 1, 2 μg as indicated) supplemented with empty vector to a total of 3 μg DNA. Luciferase activity, measured 24 h post transfection, is given in arbitrary units, error bars indicate SD, **P < 0.01, *** P < 0.005 in t-test.

**Figure 4:**
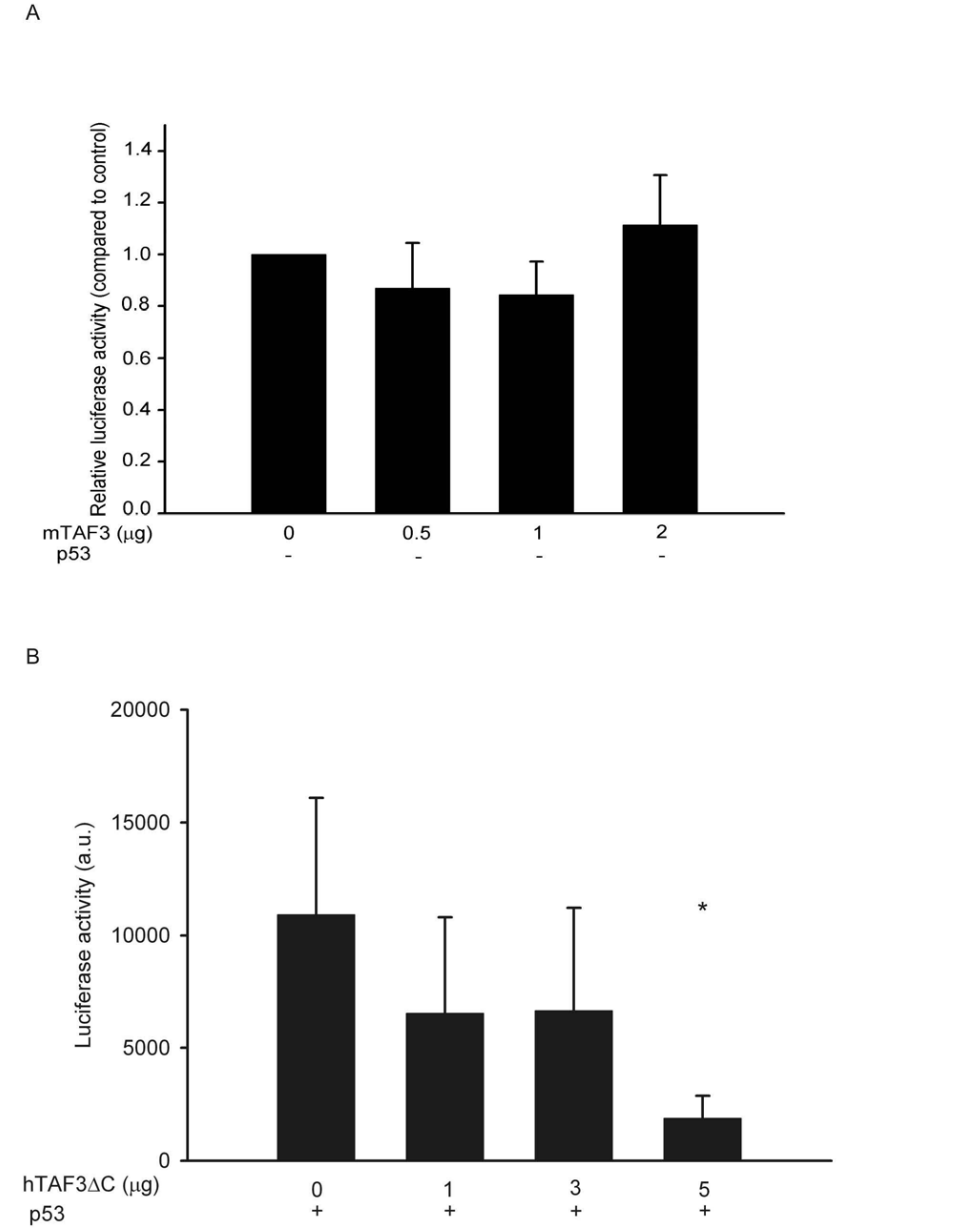
**Inhibitory activity of TAF3 is specific for p53 and it does not require the PHD finger motif**. (A) TAF3 has no effect on CMV enhancer and promoter activity. HeLa cells were co-transfected with pCDNA-luciferase reporter construct, plasmid overexpressing p53 (+), and the indicated amounts of plasmid overexpressing mTAF3 (0, 0.5, 1, 2 μg) supplemented to a total of 3 μg with vector DNA. Luciferase activity was measured 24 h post transfection and it is given relative to vector-transfected control. (B) Lack of the C terminal PHD finger motif of TAF3 has no effect on its p53-inhibitory activity. HeLa cells were co-transfected with a plasmid overexpressing p53, the p53-responsive luciferase reporter as in figure 3, plasmid overexpressing hTAF3ΔC (0, 1, 3, 5 μg) and empty vector up to a total amount of 6 μg DNA. Luciferase activity measured 24 h post transfection is given in arbitrary units, error bars indicate SD, * P < 0.05 in t-test.

To determine the functional significance of the binding of TAF3 to p53, we studied the effect of TAF3 overexpression on the transactivation activity of p53. When we transfected U2OS cells, which express wild type level of p53, with a luciferase reporter construct containing the p53-responsive element from the human *mdm2 *gene, we detected high luciferase activity (Fig. 3A, bar 0). When we co-transfected increasing amounts of a plasmid overexpressing mTAF3, we observed a dramatic (up to 100 fold) reduction in luciferase activity (Fig. 3A), indicating that TAF3 severely inhibits transcription activation by p53. To exclude that this phenomenon is cell type specific, we co-transfected HeLa cells with the same luciferase reporter as before together with a plasmid overexpressing p53 (since HeLa cells contain only very limited amount of p53) and a plasmid overexpressing mTAF3. Overexpression of TAF3 inhibited transactivation by overexpressed p53 in HeLa cells as well (Fig 3B). Note however, that the effects observed by using overexpressed p53 were less dramatic, as we saw only a 45% reduction in reporter activity (Fig 3B). We wanted to further determine if the effect of TAF3 on transcription is specific to p53 or if it is a general inhibition of transcription. When we co-transfected a luciferase reporter driven by the cytomegalovirus (CMV) promoter with increasing amounts of mTAF3, we found that TAF3 had no effect on promoter activity (Figure 4A). Thus, overexpression of TAF3 strongly and specifically inhibits the transcriptional activity of endogenous p53.

The PHD domain of TAF3 has been found to interact with tri-methylated histone H3 [33], thus it seems to be important for recognizing transcriptionally active genes. Therefore, we wanted to determine if the PHD domain of TAF3 is essential for its inhibitory role on p53. We cloned the human TAF3 lacking the PHD domain (designated hTAF3ΔC) from HeLa cDNA library. In co-transfection experiments, hTAF3ΔC inhibited transactivation by p53 in HeLa cells (Fig. 4B). The degree of inhibition using hTAF3ΔC or full length mTAF3 was comparable (40% inhibition of transcription using 1 microgram of hTAF3ΔC versus 30% inhibition using 1 microgram of full length mTAF3, compare Fig. 4B and 3B). Therefore, we conclude that the PHD domain of TAF3 is not essential for its role as a negative regulator of p53 transcriptional activity.

### Overexpression of TAF3 decreases the p53 protein level but has no effect on the p53 mRNA level

**Figure 5:**
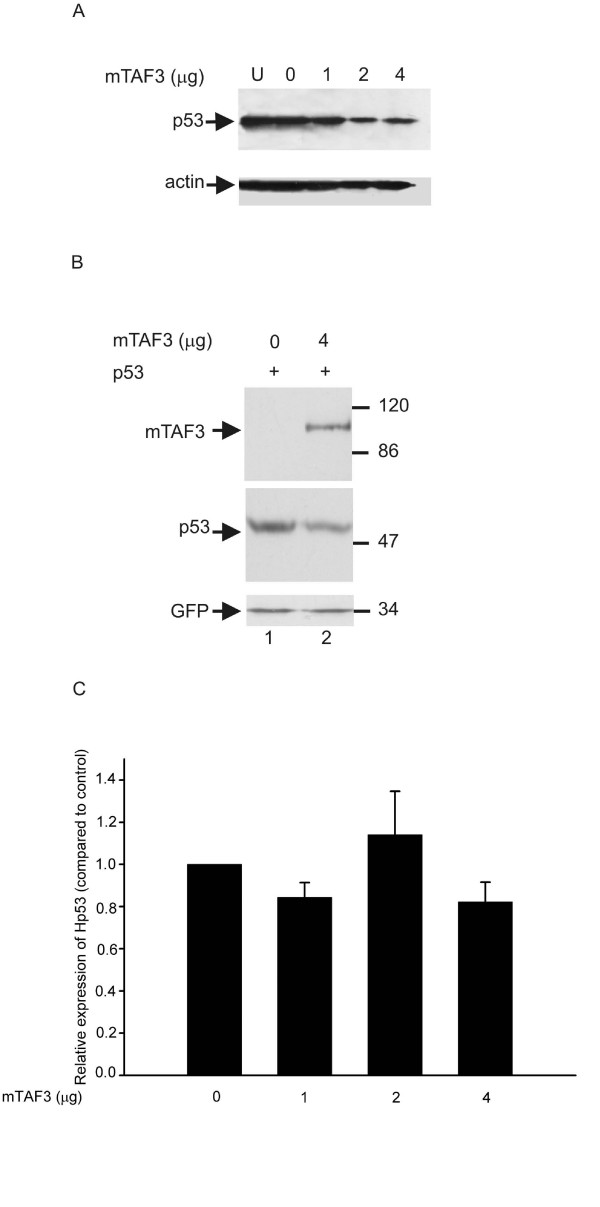
**Overexpression of TAF3 decreases the level of p53 protein but not p53 mRNA**. (A) U2OS cells were untransfected (U) or co-transfected with various amounts of plasmid expressing mTAF3 (0, 1, 2, 4 μg as indicated above the panel), and empty vector to a total of 4 μg DNA. p53 protein level in the cells was examined by Western blotting and the same blot was immunoblotted with anti-actin antibody for internal control. (B) HeLa cells were co-transfected with p53 expression construct, GFP-expression construct for control, and empty vector (0) or 4 μg of plasmid expressing mTAF3 (4). Cell lysates were prepared 24 h after transfection and analysed by Western blotting using anti-TAF3, anti-p53, or anti-GFP antibodies as indicated. (C) U2OS cells were co-transfected with various amounts of plasmid expressing mTAF3 (0, 1, 2, 4 μg) and empty vector to a total of 4 μg DNA. p53 and for internal control, GAPDH mRNA level was examined by QPCR, p53 mRNA level was normalized to GAPDH mRNA level in each sample, and expressed relative to normalized p53 mRNA level in vector-transfected control.

To further investigate the mechanism of inhibition of p53 transcriptional activity, we wanted to determine if TAF3 has an effect on the level of p53 protein. Transfection of increasing amounts of TAF3 into U2OS cells resulted in reduced level of endogenous p53, but no change in the level of endogenous β-actin was observed (Figure 5A). To exclude the cell type specificity of this phenomenon, we co-transfected p53 and GFP with or without TAF3 into HeLa cells. Here too, we saw down-regulation of p53, but not GFP, protein levels upon overexpression of TAF3 (Figure 5B), indicating that TAF3 decreases the p53 protein level. In HeLa cells the decrease in the level of p53 was comparable to the inhibition in the transcriptional activity of p53, however, in U2OS cells the reduction in the level of the p53 protein was not as dramatic as the reduction in the transcriptional activity of p53 (compare Figures 5 and 3) suggesting that down-regulation of p53 protein level may not solely be responsible for decreased transcriptional activity of p53.

We wanted to further determine if reduced level of the p53 protein is a result of decreased level of the p53 mRNA. We measured the level of endogenous p53 mRNA in U2OS cells transfected with empty vector or increasing amounts of TAF3 by performing reverse transcription and QPCR. We found no effect of TAF3 overexpression on the level of p53 mRNA (Figure 5C) suggesting that TAF3 does not influence the mRNA expression of p53, but rather affects the protein expression or turnover of p53. Thus, we conclude that TAF3 may inhibit transcription activation function of p53 partly by reducing the level of p53 protein.

## Discussion

BIP2/DmTAF3 was first identified in a yeast two hybrid screen as an interacting partner of BAB1 and BAB2 (Bric à brac 1 and 2) developmentally regulated transcription factors, which are involved in pattern formation and morphogenesis in *Drosophila *[27]. The BTB/POZ domains of BAB1 and BAB2 were shown to mediate the direct interaction with BIP2/TAF3, and amino acids 859 to 1091 in the middle part of BIP2/DmTAF3 were responsible for the binding to BAB1/2. Whether the interaction between BAB1/2 and BIP2/DmTAF3 mediates transcriptional activation or repression has not been determined.

BIP2/DmTAF3 was also identified as TAF_II_155, the *Drosophila *homolog of yeast TAF3, formerly called yTAF_II_47 [28]. DmTAF3 was shown to heterodimerize with DmTAF10 and to be a component of the general transcription factor TFIID. DmTAF3 localizes to puffs and interbands on polytene chromosomes, which correspond to transcriptionally active chromatin. The mammalian homolog of DmTAF3 was designated TAF_II_140 [28] and later called TAF3 [31]. Human TAF3 was also shown to bind to TAF10 through its histone fold domain and to be a component of both TFIID and TFTC. TAF10 relies on TAF3, among other TAFs, for its nuclear import [34].

TAF3 expression is developmentally regulated, as it is strongly expressed in early spermatocytes, but it is downregulated in haploid cells [29]. Differentiation of myoblasts to myotubes involves the disruption of a "normal" TFIID complex and its replacement by a not yet well characterized TRF3(TBP2)-TAF3 complex [30]. The switching of core promoter recognition complexes during differentiation provides cells to selectively turn on one transcription program, while silencing many others, and thus it may represent a general mechanism for regulating cell type-specific terminal differentiation.

Here we identified TAF3 (*Drosophila *and mammalian) as an interacting partner of p53. The central region, amino acids 738 to 924 of DmTAF3 mediates the interaction with p53 (Fig. 1A), a region distinct from the proposed A/T hook domain (amino acids 574–586). Interestingly, several researchers have recently identified the same central region of DmTAF3 to interact with various transcription factors, such as GAGA factor (amino acids 612–1073) [35], Antennapedia (amino acids 853–1088) [36] and BAB1/2 (amino acids 859 to 1091) [27]. Although this central region of DmTAF3 shows low degree of similarity to the corresponding core region of mammalian TAF3 (amino acids 497 to 608 of murine TAF3) [28], we propose that it may serve as a transcription factor-binding domain responsible for interaction with other, yet unidentified transcription factors as well. We think that the TAF3-p53 interaction is direct and does not involve previously characterized p53 interacting components of TFIID, since we detected *in vitro *binding between bacterially expressed Dmp53 and *in vitro *translated portion of DmTAF3 (Fig. 1D). The interaction is evolutionarily conserved, since DmTAF3 can also interact with human p53 (Fig. 1C), as well as mammalian TAF3 can bind to both Dmp53 and human p53 in heterologous systems (Fig. 2). Importantly, unlike previously identified TAFs that interact with the N terminal transcription activation domain of p53 and mediate transcription activation, TAF3 interacts with the C terminal oligomerization and regulatory domain of p53 and represses p53 activity.

Elevated TAF3 expression strongly inhibits transcription activation by p53 in at least two human cell lines containing either endogenous or overexpressed p53 (Fig. 3). This effect of TAF3 is specific to p53, since we found no effect of TAF3 on transcription regulated by the CMV promoter (Fig. 4A) indicating that it does not inhibit the transcriptional activity of the factors that bind to these elements. Furthermore, in other studies TAF3 had no inhibitory effect on ATF7 [37], and it even acted as a coactivator for WDR5-dependent genes [33]. The PHD finger is a specialized form of zinc finger that is classically characterized by C4HC3 arrangement of cysteine and histidine residues involved in Zn coordination [38]. The PHD finger was shown to be essential for the coactivator activity of TAF3 [33], in contrast, we found that the PHD finger was not required for the inhibitory activity of TAF3 on p53. Although most PHD finger containing proteins were implicated in mediating interaction with chromatin [33,39,40], other reports described E3 ubiquitin ligase activity of PHD containing viral and cellular proteins [41-44]. Since we found that overexpression of TAF3 reduces p53 protein level (Fig. 5), we examined the possibility that TAF3 may act as an E3 ubiquitin ligase for p53. However, in our preliminary experiments we could not detect any ubiquitin ligase activity of TAF3 towards p53 in either *in vitro *or *in vivo *ubuiquitination assays (data not shown). Because TAF3 is a subunit of TFIID, and interacts with several additional proteins [45], it is conceivable that TAF3 recruits a ubiquitin ligase to p53 to increase its turnover. In HeLa cells the reduction in the level of p53 was comparable to the reduction seen in the transcriptional activity of p53, while in U2OS cells the decrease in the level of the p53 protein was not as dramatic as the reduction seen in the transcriptional activity of p53 (Figures 3 and 5). This latter observation suggests that down-regulation of p53 protein level by TAF3 may not solely be responsible for reduced transcriptional activity of p53, but additionally, TAF3 may directly inhibit the transcriptional activity of p53. We speculate that the additional mechanisms TAF3 employs to inhibit p53 transcriptional activity may include posttranslational modifications, such as sumoylation [46], but the exact mechanisms remain to be elucidated.

## Conclusion

Here we identify TAF3 as an evolutionarily conserved negative regulator of p53 transcription activation function. We show physical interaction between the C terminus of Dmp53 and the central region of TAF3 both in yeast two hybrid assays and *in vitro*. The interaction is evolutionarily conserved, since DmTAF3 can also interact with human p53, and mammalian TAF3 can bind to both Dmp53 and human p53. Elevated TAF3 expression results in severe specific inhibition of transcription activation by p53 in human cell lines, which may be partly due to a decrease in the level of the p53 protein.

## Methods

### Plasmid constructs

Bait plasmids pBTM116-Dmp53ΔN (amino acids 47–385), pBTM116-Dmp53ΔNΔC (amino acids 47–294) pBTM116-Dmp53C (amino acids 277–385), pBTM116-Dmp53C1 (amino acids 277–351), pBTM116-Dmp53C2 (amino acids 349–385), pBTM116-p53ΔN, pGEX4T1-Dmp53C and pXJ41-mTAF3 were described previously [28,32]. The cDNA encoding DmTAF3 amino acids 514 to 924 was cloned in frame into pET28c (Novagen). Human full length p53 was cloned in frame into pGEX6P1 vector (Amersham Biosciences) using BamHI and XhoI restriction sites. To generate pCDNAflag-hTAF3ΔC cDNA from HeLa cells was PCR-amplified using the following primers: hTAFfw AGAATTCAGTTACTCCAGGTCGTTGTTG, hTAFrev AAAGTCGACGGAAGCACTGGCAACAAAG. The resulting PCR product was cloned into pCDNAflag using EcoRI and SalI restriction sites. To construct the baculovirus expression vector for TAF3, the full length mTAF3 cDNA was excised from the pXJ41-Flag-TAF3 expression vector by EcoRI and Bam HI and inserted in the corresponding sites of the pVL1392 baculovirus expression vector. The generation of the Flag-TAF3 expressing recombinant baculovirus and the Sf9 cell infection was done under standard conditions [47].

### Yeast two-hybrid experiments

The yeast two-hybrid screen was performed following the manufacturer's recommendations using MATCHMAKER LexA Two-Hybrid System with the MATCHMAKER *Drosophila *embryonic cDNA library cloned into pACT2 (Clontech Laboratories, Inc.) as previously described [32]. Positive clones were validated by β-galactosidase colony-lift filter assay [48]. Plasmid DNA was isolated from colonies proved to be positive in both complementation of auxotrophy and β-galactosidase assays, sequenced and cDNAs identified by BLAST homology searches [49].

### *In vitro *binding assays

DmTAF3(514–924) was transcribed and translated from pET28c-DmTAF3clone5 in the presence of ^3^H leucine using the TNT T7 Qiuck-coupled transcription-translation system (Promega) following the manufacturer's instructions. pGEX4T1-Dmp53C, pGEX6P1 or pGEX6P1-p53 was introduced into *E. coli *strain BL21, protein expression was induced by treatment with IPTG over night at 37°C. Expressed GST-Dmp53C or GST was bound to glutathione sepharose high performance beads (Amersham Biosciences) according to the manufacturer's instructions, incubated with labelled DmTAF3(514–924) and interaction was analysed as described before [32].

Sf9 cell extract overexpressing murine TAF3 by baculoviral expression system [34] was incubated with bacterially expressed GST, GST-Dmp53C or GST-p53 bound to glutathione sepharose beads over night at 4°C as previously described. Beads were washed with various concentrations of KCl as indicated in Figure 2B, then bound proteins were analysed with SDS-PAGE and Western blotting using anti-TAF3 antibody 39TA1C7 [28].

Full length human p53, *Drosophila melanogaster *ADA2A and firefly luciferase as control were transcribed and translated in the presence of ^35^S methionine using the TNT T7 Quick-coupled transcription-translation system (Promega) following the manufacturer's instructions. Sf9 cell extract overexpressing murine TAF3 was incubated with the indicated (Fig. 2C) single labelled protein or p53 and luciferase together, and complexes were immunoprecipitated using anti-TAF3 antibody 39TA2F5 as described before [34]. Precipitated complexes were washed 3 times with PBS and once with PBS supplemented with 200 mM KCl. Labelled proteins were detected by SDS-PAGE and fluorography (using Amplify Fluorographic Reagent from Amersham Biosciences).

### Cell culture, transfection and luciferase reporter assays

HeLa human cervical carcinoma, U2OS human osteosarcoma and 293T human embryonal kidney cells were maintained in Dulbecco's modified Eagle's medium, supplemented with 5% (for HeLa) or 10% (for U2OS and 293T) foetal bovine serum in 5% CO2 incubators at 37°C. For Western blotting and QPCR U2OS cells were transfected on 60 mm dishes using ExGen transfection reagent (Fermentas) with the amount of pXJ41-mTAF3 indicated on the figures, supplemented to a total of 4 μg with empty vector DNA. For Western blotting HeLa cells were transfected with 0.5 μg pCDNA3p53Pro, 0.5 μg pEGFP and the indicated amount of pXJ41-mTAF3 supplemented to 5 μg with empty vector. For luciferase reporter assays cells were transfected in 6-well plates with 0.5 μg pGL2NaLuc reporter (p53-responsive construct from the *mdm2 *gene) or pCDNA3-luc reporter and 0.5 μg pCDNA3p53Pro where indicated and with the amount of pXJ41-mTAF3 or pCDNA3flag-hTAF3ΔC indicated on the figures, supplemented to a total of 3 or 6 μg with empty vector DNA, respectively. Twenty-four hours after transfection cells were lysed with Reporter Lysis Buffer (Promega), and luciferase reporter assay was performed using Luciferase Assay System as recommended by the manufacturer (Promega). As a control for equal cell number after transfection, protein concentration of the samples was measured using Bradford reagent (Bio-Rad). Mean and standard deviation of luciferase activity data was calculated from three separate experiments, and P-values were calculated using paired t-test.

### Immunoprecipitation and Western blotting

For co-immunoprecipitation assay 293T cells were transfected on 14 cm dish using JetPEI (Q-Bio Gene) with 7 μg pCDNA3p53Pro and 7 μg pXJ41-mTAF3 or empty vector DNA. Cells were lysed 48 h after transfection and immunoprecipitation of TAF3-complexes was performed as described before using anti-TAF3 39TA1C7 antibody [28]. Western blotting and immunodetection was performed using anti-human p53 DO-1 (Santa Cruz Biotechnology) or DO-7 (Dako Cytomation), anti-actin20-33 (Sigma), anti-GFP (Invitrogen), anti-TAF3 39TA1C7 [28] primary antibodies, and anti-mouse-HRP (Dako Cytomation) or anti-kappa light chain-HRP (for detection of p53 after co-IP) secondary antibodies.

### Quantitative RT-PCR

Total RNA was isolated with Qiagen RNeasy mini kit (Qiagen Inc.) according to the manufacturer's instructions. First-strand cDNA was synthesized from 2 μg total RNA with random hexamer primers using TaqMan Reverse Transcription Reagent (Applied Biosystems). PCR reactions were carried out in duplicates in an ABI Prism 7500 real-time PCR system using SYBR-Green PCR Master Mix and the following primers: hp53For CCCTTCCCAGAAAACCTACC, hp53Rev CTCCGTCATGTGCTGTGACT, GAPDHfor ACCTGACCTGCCGTCTAGAA, GAPDHrev TCCACCACCCTGTTGCTGTA. The C_t _value for each studied mRNA was normalized to the GAPDH internal control and the change of expression levels of the examined genes were calculated by the ΔΔCt method.

## Abbreviations

DBD: DNA binding domain; DmTAF3: *Drosophila melanogaster *TAF3; GAPDH: glyceraldehyde phosphate dehydrogenase; GFP: green fluorescent protein; GST: glutathione S-transferase; mTAF3: *Mus musculus *TAF3; PBS: phosphate buffered saline; PHD: plant homeodomain; QPCR: quantitative real time polymerase chain reaction; rpb4 and 7: RNA polymerase II subunit 4 and 7 gene; SD: standard deviation; TAF3: TATA binding protein associated factor 3; TBP: TATA binding protein; Y2H: yeast two hybrid.

## Authors' contributions

OB carried out most of the experiments and participated in the writing of the manuscript. ZU carried out the QPCR experiments, NP carried out the *in vitro *pull-down experiments and ZN carried out the co-immunoprecipitations from cells. LT participated in the design and coordination of the study and helped to draft the manuscript. IMB and EB conceived the study, designed experiments and drafted the manuscript. All authors read and approved the final manuscript.
